# The Cognitive, Behavioral, and Emotional Aspects of Eating Habits and Association With Impulsivity, Chronotype, Anxiety, and Depression: A Cross-Sectional Study

**DOI:** 10.3389/fnbeh.2019.00204

**Published:** 2019-09-06

**Authors:** Carla Aoun, Lynn Nassar, Stéphanie Soumi, Nada El Osta, Tatiana Papazian, Lydia Rabbaa Khabbaz

**Affiliations:** ^1^Laboratoire de Pharmacologie, Pharmacie Clinique et Contrôle de qualité des médicaments, Faculty of pharmacy, Saint-Joseph University of Beirut, Beirut, Lebanon; ^2^Department of Nutrition, Faculty of Pharmacy, Saint-Joseph University of Beirut, Beirut, Lebanon; ^3^Department of Public Health, Faculty of Medicine, Saint-Joseph University, Beirut, Lebanon; ^4^Department of Prosthodontics, Faculty of Dental Medicine, Saint-Joseph University, Beirut, Lebanon

**Keywords:** eating habits, cognitive restraint, uncontrolled eating, emotional eating, impulsivity, chronotype, anxiety, university students

## Abstract

**Background and objectives:** Understanding behavioral issues associated with eating would provide important insight into obesity development and possibly procure ways to prevent its occurrence or to treat it. This study's objectives were to examine links between cognitive, behavioral, and emotional aspects of eating habits and chronotype, impulsivity, anxiety, and depression among university students.

**Subjects and methods:** The following questionnaires were used: TFEQ-R 18, UPPS-short, HADS, and MEQ. All participants gave their informed written consent prior to enrolment.

**Results:** Among females, increased BMI was associated to uncontrolled eating and emotional eating, while in males, BMI was associated to emotional eating only. In males, no associations of BMI with impulsivity were found while in females they were present. Chronotype scores were positively correlated to cognitive restraint and negatively to uncontrolled eating among males. No associations were found for females. CR was lower among females with higher depression scores, while higher anxiety scores were associated to UE among males.

**Conclusions:** This was a cross-sectional study of three cognitive and emotional domains related to eating habits among university students (young adults). Results showed significant correlations between BMI, TFEQ-R18 scores, impulsivity and anxiety or depression. Future studies should replicate findings in samples of individuals with different aspects of eating disorders such as binge eating disorder, food addiction or bulimia nervosa.

## Introduction

Obesity is one of the most prevalent public health problems but is often ignored (Singh et al., 2017). Numerous factors at the individual, interpersonal, environmental, and macrosystem levels contribute to obesity (Banna et al., 2018). Understanding behavioral eating issues would provide important insight into obesity development and possibly procure ways to prevent its occurrence or to treat it. One of the tools used to examine disorders in eating behaviors related to development of obesity is the Three-Factor Eating Questionnaire (TFEQ) (de Lauzon et al., 2004). It was initially developed to assess cognitive restraint, disinhibition, and susceptibility to hunger in adults (Stunkard and Messick, 1985). The TFEQ, which was first created by Stunkard and Messick (1985), is a widely used instrument in eating behavior researches all over the world. The TFEQ-R18 is a shortened, revised version of the TFEQ questionnaire with three subscales, where disinhibition and hunger scales were grouped in a single subscale and labeled as uncontrolled eating (UE); The Cognitive Restraint (CR) scale was shortened, and a third subscale was added including three items tagged as emotional eating (EE) (Provencher et al., 2003; Hays and Roberts, 2008).

CR or restricting eating to control body weight is characterized as the cognitive and self-imposed limitation of food ingestion to control weight (Preedy et al., 2011). However, disinhibited eating could happen among individuals with high cognitive restraint under stress (Zigmond and Snaith, 1983; Boutelle et al., 2010).

The UE behavior is a tendency to lose control regarding food when an individual feels hungry or when external cues (e.g., very palatable food) are present. This could happen even when physiological hunger is absent (Tholin et al., 2005). Finally, EE is the tendency to eat in response to negative mood states and emotional stress (Gibson, 2012). EE is considered a common dimension of all eating disorders (Rotella et al., 2015) and is more frequent among women than men (Kemp et al., 2011). People who present an increased CR and a decreased EE and/or UE achieve better results when following weight-loss programs (Keränen et al., 2009; Konttinen et al., 2009).

In addition to eating behavior, multiple studies shows that sleeping habits have an impact on regulation of body weight (Chaput et al., 2011). Short sleep duration and poor sleep quality are major factors linked to weight and eating pattern. A longitudinal study of a large sample of adults reported higher food consumption in the event of sleep deprivation (Chaput et al., 2011) leading to an increase in stress-related eating (Bond et al., 2001).

Chronotype is a circadian typology that splits individuals into morning, intermediate or evening types, each with its own properties and predispositions to several diseases. The disparity between chronotype groups is an important factor that affects sleep and sleep patterns (Adan et al., 2012; Song et al., 2018). Night-type individuals exhibit a social jet lag that is defined by a gap between the social clock and the endogenous circadian clock, leading to chronic sleep loss, metabolic and psychological modification, including cardiovascular diseases, type 2 diabetes, depressive symptoms, and emotional eating (Haffen, 2009; Konttinen et al., 2014). On the other hand, a positive association is shown between morning chronotype and dietary restriction (Schubert and Randler, 2008).

Another factor contributing to eating behavior is impulsivity: some dimensions of impulsivity have been implicated in binge eating and more specifically in exaggerated food consumption with loss of control (Espel et al., 2017). Impulsivity is defined as a general tendency to have rapid or even unforeseen reactions to external or internal stimuli regardless of the consequences of these actions on oneself or others (Jasinska et al., 2012). Depression, anxiety, chronotype and impulsivity are closely related (Caci et al., 2005; Moustafa et al., 2017). This study's objectives were to examine links between cognitive, behavioral, and emotional aspects of eating habits and chronotype, impulsivity, anxiety, and depression among young adults (university students). To the best of our knowledge, no previous study has assessed these associations.

## Materials and Methods

### Ethical Considerations

The ethics committee of Saint-Joseph University (Ref USJ-2017-81) approved the protocol of this study. Informed written consent was obtained from all participants in the study.

### Survey Procedure and Sampling

This is a cross-sectional questionnaire-based survey conducted among Lebanese university students (Grand Beirut universities: USJ, Lebanese university, USEK, ALBA, NDU, LAU, SAGESSE), from October 2017 till March 2018. Inclusion criteria were: students ≥18 years, willingly participating in the study. Exclusion criteria were the presence of any cognitive deficit or other chronic diseases. A random number table was used to randomly select students within each university, in order to ensure the representativeness of the sample. The random selection was proportional to students number in each university. The selected students were approached at the end of their courses, before leaving the classroom, by two trained research assistants. Out of 580 students approached (56% females−44% males), 400 agreed to participate. In the final sample, 81% of females approached agreed to participate while only 53.7% of males approached did.

### Data Collection

Data were collected during a face-to-face interview, using a survey tool (self-administered and standardized) based on internationally reliable and validated questionnaires, namely the TFEQ-R 18, the UPPS-short, the HADS, and the MEQ. The duration of interviews ranged from 15 to 25 min.

### Measures

#### Participants

Socio-demographic characteristics of the participants were collected. The crowding index (number of people living in the same house divided by the number of rooms in the house excluding the kitchen and bathrooms) was calculated. Caffeine intake was assessed with two questions: number of cups of coffee/day and number of units of any beverage containing caffeine (mainly soft drinks containing caffeine).

#### Eating Habits Assessed With the Three-Factor Eating Questionnaire Revised 18-Item Version (TFEQ-R 18)

Originally consisting of 51 items, Karlsson et al. (2000) developed a reduced version of the TFEQ-R18 (Three-Factor Eating Questionnaire Revised 18-item version). The reduced version assesses three main eating behaviors: emotional eating (EE), uncontrolled eating (UE), and cognitive restraint (CR). The components of eating behavior assessed by this questionnaire are related to energy and macronutrient intake or intake of certain types of foods. It is based on a four-point answer scale (absolutely true, mostly true, absolutely wrong, and mostly false). Responses to each of the 18 points have a score range between 1 and 4 and the scores are added (de Lauzon et al., 2004). The Cronbach alpha was 0.726.

#### Impulsivity Assessed With the Impulsive Behavior Scale (UPPS-P Short Version)

The UPPS scale was originally developed by Whiteside and Lynam (2003). Four impulsivity traits were included in the original version of the scale: lack of premeditation, negative urgency, sensation seeking, and lack of perseverance (Jasinska et al., 2012; Booth et al., 2018). Impulsive action under extreme positive emotions were not well-conceptualized in this scale. Therefore, Cyders and colleagues created a scale of positive urgency, which was later incorporated into the UPPS-P scale and in the shorter version, the UPPS-P short version that we used in this study (Cyders et al., 2014). Higher scores indicate higher impulsivity. The Cronbach alpha was 0.843.

#### Evaluation of Anxiety and Depression With the Hospital Anxiety and Depression Scale (HADS)

The HADS is an effective instrument for evaluating anxiety and depressive disorders and was validated by Zigmond and Snaith (1983). Fourteen items are included in the scale. Each item is rated on a scale from 0 to 3. The HADS is divided into two subscales: HADS-A with seven items built to the clinical criteria for diagnosis of anxiety symptoms and HADS-D, with seven other items built to the clinical criteria for diagnosis of depression symptoms. Each subscale score is calculated by summing the points of the items included in the subscale and the value range is 0–21. The interpretation of the obtained score is as follows: ≤7: absence of anxiety/depression; 8–10: borderline abnormal; 11–21: severe anxiety/depression. A total HADS score can also be calculated. The Cronbach alpha was 0.771.

#### Evaluation of the Chronotype With the Morningness-Eveningness Questionnaire (MEQ)

The Morningness-Eveningness Questionnaire (MEQ) is a widely-used international questionnaire validated by Horne and Ostberg (1976). This questionnaire is composed of 19 items. It is self-administered and measures the person's peak alertness/sleepiness (morning or evening). MEQ consists of 19 questions allowing to calculate a total score between 16 and 86; scores ≤30 indicate definite evening type, 31–41 indicate moderate evening type, 42–58 intermediate type, 59–69 moderate morning type, and 70–86 definite morning type. The Cronbach alpha was 0.784.

#### Data Analysis

##### Statistical analysis

The statistical analyses were performed with SPSS software for Windows (version 24.0, Chicago, IL, USA). A significance level of 0.05 was set. Means and standard deviations were calculated for continuous variables and percentages for categorical variables. The normality distribution of continuous variables was tested with Kolmogorov-Smirnov tests. Chi-square independence tests and Fisher Exact tests were used to examine the relationship between categorical variables. Pearson and Spearman correlation coefficients were used to evaluate the association between continuous variables. In step one, univariate analyses were carried out with the Student's *t*-test or its equivalent non-parametric Mann-Whitney test and analysis of variance (ANOVA) or its equivalent non-parametric Kruskal-Wallis test. Univariate followed by multivariate analyses were performed to assess the association between explanatory variables and eating habits dimensions.

Each of cognitive, behavioral and emotional eating was the dependant variables of the study. Impulsivity, anxiety and depression were the explanatory independent variables.

Candidates for the multivariate model, according to the Enter method were the independent variables that showed associations with a *p* <0.200 in univariate analyses. Collinearity among independent variables was also examined and we excluded variables highly correlated from the model. Interaction between each component of impulsivity and anxiety/depression was also tested when introduced in the same multivariate model. The interaction results between impulsivity and anxiety/depression were not statistically significant (*p* > 0.05).

## Results

The socio-demographic characteristics of the participants are summarized in Table 1. 400 students were included (65.8% females), with a mean age of 20.39 (SD = 1.83).

**Table 1:** Sociodemographic characteristics of the participants.

			***N***		**Percentage**
**GENDER**					
Male			137		34.2
Female			263		65.8
**LIVING**					
Alone			16		4.0
In couple			2		0.5
With family			381		95.2
Missing values			1		0.2
BMI CATEGORIZED^*^					
**Males**					
Underweight			8		5.8
Normal			86		62.8
Overweight			42		30.7
Obese			1		0.7
**Females**					
Underweight			42		16.1
Normal			199		76.2
Overweight			17		6.5
Obese			3		1.1
**ECONOMIC STATUS OF THE STUDENT**					
Not working			342		85.5
Working			55		13.7
Missing values			3		0.8
**SMOKING STATUS**					
No			280		70.0
Ex-smoker			21		5.2
Yes			81		20.2
Missing values			18		4.5
**ALCOHOL INTAKE**					
No			90		22.5
Occasionally			214		53.5
≤ once per week			50		12.5
> once per week			35		8.8
Missing values			11		2.8
**COFFEE**					
No			172		43.0
1–2 cups/day			186		46.5
>2 cups/day			38		9.5
Missing values			4		1.0
	***N***	**Minimum**	**Maximum**	**Mean**	**Standard deviation**
Age	400	18	30	20.39	1.83
Crowding index	399	0.0	4.0	0.95	0.44
BMI^*^	398	16.0	32	22.06	3.12
Males	137	16.1	32.2	23.78	2.99
Females	263	16.0	30.5	21.15	2.78

* *Significant difference between males and females (p <0.001)*.

BMI was analyzed in two different ways: as continuous variable and was also categorized according to the World Health Organization (WHO) cut-off points (underweight < 18.5, normal 18.5–24.9, overweight 25–29.9, and obese > 30 kg/m^2^) (WHO, 1995).

The average scores relative to the questionnaires, according to gender, are summarized in Table 2. Among eating habits, emotional eating component was significantly different between males and females (*p* = 0.038). Impulsivity facets and chronotype did not show gender differences. Anxiety scores were significantly higher among females and depression scores higher among males (*p* = 0.000 and 0.039, respectively).

**Table 2:** Eating habits, impulsivity, chronotype, anxiety, and depression: average scores, standard deviation (SD) and percentages.

		**Men**	**Women**	**Total**	***p***
Eating habits (TFEQ-R 18)	Cognitive restraint	12.99 ± 3.244	13.19 ± 3.566	13.12 ± 3.456	0.596
	Uncontrolled eating	21.61 ± 5.524	20.80 ± 5.448	21.08 ± 5.481	0.164
	Emotional eating	6.62 ± 2.346	7.16 ± 2.549	6.98 ± 2.491	0.038
Impulsivity (UPPS)	Negative urgency	9.82 ± 3.001	9.91 ± 3.045	9.88 ± 3.026	0.766
	Positive urgency	10.69 ± 2.789	10.90 ± 2.730	10.82 ± 2.748	0.466
	Lack of premeditation	7.42 ± 2.637	7.36 ± 2.795	7.38 ± 2.738	0.820
	Lack of perseverance	7.42 ± 2.785	7.24 ± 3.107	7.30 ± 2.999	0.571
	Seeking sensations	11.71 ± 2.883	11.30 ± 3.212	11.44 ± 3.106	0.209
Anxiety and depression (HADS)	Anxiety	7.77 ± 3.824	9.24 ± 3.829	8.74 ± 3.886	0.000
	Depression	6.96 ± 3.705	6.24 ± 3.124	6.48 ± 3.348	0.039
	HADS	14.73 ± 6.665	15.48 ± 6.121	15.22 ± 6.314	0.263
Chronotype (MEQ)	Chronotype	47.39 ± 9.218	47.70 ± 9.306	47.60 ± 9.266	0.746
	Definite evening type	7 (5.1%)	6 (2.3%)	13 (3.2%)	
	Moderate evening type	28 (20.4%)	57 (21.7%)	85 (21.2%)	0.568
	Intermediate type	87 (63.5%)	169 (64.3%)	256 (64.0%)	
	Moderate morning type	15 (10.9%)	29 (11.0%)	44 (11.0%)	
	Definite morning type	0 (0.0%)	2 (0.8%)	2 (0.5%)	

Tables 3, 4 show the correlations found between the different components of the questionnaires and the continuous sociodemographic factors among males and females. BMI was significantly correlated to cognitive restraint and emotional eating in both gender; however, no significant correlation was detected between BMI and uncontrolled eating among males. BMI was also significantly and positively correlated to lack of premeditation among males as well as to higher depression scores among females and higher anxiety scores among males.

**Table 3:** Associations between the different components of the questionnaires and the continuous socio-demographic characteristics of male participants (*N* = 400).

		**Age**	**Crowding index**	**BMI**	**Alcohol**	**Coffee**
**Tfeq-R 18**						
Cognitive restraint	Correlation coefficient	−0.024	−0.095	**0.207**	−0.095	−0.005
	*p*-value	0.777	0.272	**0.015**	0.279	0.956
	*N*	137	136	**137**	133	137
Uncontrolled eating	Correlation coefficient	**−0.251**	−0.001	0.153	**0.177**	0.125
	*p*-value	**0.003**	0.990	0.074	**0.042**	0.145
	*N*	**137**	136	137	**133**	137
Emotional eating	Correlation coefficient	−0.024	−0.008	**0.192**	0.075	0.075
	*p*-value	0.781	0.927	**0.024**	0.393	0.387
	*N*	137	136	**137**	133	137
**Upps**						
Negative urgency	Correlation coefficient	0.079	0.086	0.128	0.008	−0.057
	*p*-value	0.357	0.322	0.136	0.924	0.510
	*N*	137	136	137	133	137
Positive urgency	Correlation coefficient	−0.125	0.023	0.087	−0.109	−0.036
	*p*-value	0.146	0.791	0.314	0.210	0.675
	*N*	137	136	137	133	137
Lack of premeditation	Correlation coefficient	−0.040	**0.368**	**0.194**	0.022	−0.023
	*p*-value	0.647	**0.000**	**0.023**	0.797	0.793
	*N*	137	**136**	**137**	133	137
Lack of perseverance	Correlation coefficient	−0.122	**0.334**	0.123	−0.097	−0.040
	*p*-value	0.157	**0.000**	0.155	0.268	0.644
	*N*	136	**135**	136	132	136
Seeking sensations	Correlation coefficient	−0.030	0.056	−0.065	**0.176**	0.042
	*p*-value	0.730	0.517	0.450	**0.042**	0.626
	*N*	137	136	137	**133**	137
**Hads**						
Anxiety	Correlation coefficient	−0.008	−0.007	**0.175**	−0.148	0.099
	*p*-value	0.928	0.935	**0.040**	0.089	0.252
	*N*	137	136	**137**	133	137
Depression	Correlation coefficient	0.049	0.015	0.074	0.078	0.130
	*p*-value	0.568	0.859	0.392	0.371	0.131
	*N*	137	136	137	133	137
HADS	Correlation coefficient	0.023	0.005	0.142	−0.043	0.129
	*p*-value	0.791	0.958	0.099	0.627	0.134
	*N*	137	136	137	133	137
Chronotype	Correlation coefficient	**0.215**	**−0.221**	0.114	−0.110	−0.060
	*p*-value	**0.012**	**0.010**	0.184	0.207	0.486
	*N*	**137**	**136**	137	133	137

Spearman and Pearson correlation was used.

*Bold mean that the results are significant*.

**Table 4:** Associations between the different components of the questionnaires and the continuous socio-demographic characteristics of female participants (*N* = 400).

		**Age**	**Crowding index**	**BMI**	**Alcohol**	**Coffee**
**Tfeq-R 18**						
Cognitive restraint	Correlation coefficient	0.070	−0.052	**0.170**	0.008	0.063
	*p*-value	0.261	0.404	**0.006**	0.898	0.316
	*N*	263	263	**261**	256	259
Uncontrolled eating	Correlation coefficient	0.056	−0.001	**0.184**	0.054	−0.013
	*p*-value	0.362	0.981	**0.003**	0.386	0.835
	*N*	263	263	**261**	256	259
Emotional eating	Correlation coefficient	0.098	0.100	**0.241**	0.007	−0.019
	*p*-value	0.111	0.106	**0.000**	0.910	0.764
	*N*	263	263	**261**	256	259
**Upps**						
Negative urgency	Correlation coefficient	0.034	0.075	0.047	−0.094	**0.148**
	*p*-value	0.584	0.227	0.452	0.135	**0.017**
	N	263	263	261	256	**259**
Positive urgency	Correlation coefficient	−0.006	**0.125**	−0.013	0.008	**0.126**
	*p*-value	0.917	**0.043**	0.837	0.898	**0.044**
	*N*	263	**263**	261	256	**259**
Lack of premeditation	Correlation coefficient	0.063	0.070	0.012	−0.013	**0.173**
	*p*-value	0.311	0.259	0.849	0.831	**0.005**
	*N*	263	263	261	256	**259**
Lack of perseverance	Correlation coefficient	0.000	0.055	0.038	−0.068	0.053
	*p*-value	0.997	0.372	0.544	0.278	0.397
	*N*	263	263	261	256	259
Seeking sensations	Correlation coefficient	−0.035	0.047	−0.038	**0.166**	0.035
	*p*-value	0.572	0.444	0.543	**0.008**	0.576
	*N*	263	263	261	**256**	259
**Hads**						
Anxiety	Correlation coefficient	**0.161**	0.053	0.102	−0.038	**0.213**
	*p*-value	**0.009**	0.393	0.102	0.541	**0.001**
	*N*	**263**	263	261	256	**259**
Depression	Correlation coefficient	0.061	0.067	**0.128**	−0.029	0.003
	*p*-value	0.322	0.279	**0.039**	0.643	0.965
	*N*	263	263	**261**	256	259
HADS	Correlation coefficient	**0.132**	0.067	**0.129**	−0.039	**0.134**
	*p*-value	**0.033**	0.277	**0.038**	0.536	**0.031**
	*N*	**263**	263	**261**	256	**259**
**Chronotype**						
Chronotype	Correlation coefficient	**0.121**	**0.147**	0.040	**−0.193**	0.052
	*p*-value	**0.049**	**0.017**	0.520	**0.002**	0.402
	*N*	**263**	**263**	261	**256**	259

Spearman and Pearson correlation was used.

*Bold mean that the results are significant*.

Age was significantly and positively correlated to the chronotype score in both gender. Crowding index was significantly and positively associated several impulsivity facets. As for alcohol, increased intake was associated with higher uncontrolled eating scores among males and higher seeking sensations scores in both gender. Coffee intake was associated with higher anxiety scores as well as higher lack of premeditation, positive and negative urgency among females.

Table 5 shows the association between the three components of eating habits and categorized BMI (according to gender). Tables 6, 7 present the correlations between the 3 domains of the eating habits questionnaire and the other questionnaires among females and males. Multiple significant correlations were seen between the eating habits and impulsivity (especially among females), while anxiety was significantly associated to eating habits in males.

**Table 5:** Association between eating habits and categorized BMI in males and females.

	**Underweight**	**Normal**	**Overweight**	**Obese**	***p*-value**
**Cognitive restraint**					
Male	12.75 ± 3.808	12.62 ± 3.444	13.88 ± 2.549	10.00	0.157
Female	12.17 ± 3.499	13.48 ± 3.614	12.47 ± 2.787	11.00 ± 2.000	**0.038**
**Uncontrolled eating**						
Male	18.62 ± 3.021	21.50 ± 5.710	22.19 ± 5.316	30.00	0.162
Female	19.64 ± 4.089	20.81 ± 5.558	23.53 ± 6.336	23.33 ± 7.234	0.079
**Emotional eating**						
Male	5.38 ± 1.408	6.57 ± 2.262	6.83 ± 2.498	12.00	**0.046**
Female	6.57 ± 2.539	7.16 ± 2.487	8.53 ± 2.809	8.67 ± 2.517	**0.041**

*Bold mean that the results are significant*.

**Table 6:** Correlations observed between the three aspects of eating habits and impulsivity, chronotype, anxiety, and depression among male participants.

		**CR**	**UE**	**EE**	**Negative urgency**	**Positive urgency**	**Lack of premeditation**	**Lack of perseverance**	**Seeking sensation**	**Anxiety**	**Depression**	**HADS**
Negative urgency	Coefficient	0.048	0.110	**0.188**	1							
	*p*-value	0.576	0.202	**0.028**								
	*N*	137	137	**137**	137							
Positive urgency	Coefficient	0.024	0.063	**0.174**	**0.431**	1						
	*p*-value	0.780	0.468	**0.042**	**0.000**							
	*N*	137	137	**137**	**137**	137						
Lack of premeditation	Coefficient	−0.093	0.102	−0.032	**0.461**	**0.252**	1					
	*p*-value	0.278	0.234	0.710	**0.000**	**0.003**						
	*N*	137	137	137	**137**	**137**	137					
Lack of perseverance	Coefficient	−0.105	0.076	−0.053	**0.316**	0.033	**0.624**	1				
	*p*-value	0.223	0.378	0.542	**0.000**	0.704	**0.000**					
	*N*	136	136	136	**136**	136	**136**	136				
Seeking sensations	Coefficient	−0.079	0.141	0.069	0.109	**0.178**	0.035	−0.058	1			
	*p*-value	0.360	0.099	0.420	0.203	**0.038**	0.687	0.501				
	*N*	137	137	137	137	**137**	137	136	137			
Anxiety	Coefficient	**0.180**	**0.193**	**0.181**	**0.327**	**0.259**	**0.316**	**0.206**	−0.038	1		
	*p*-value	**0.035**	**0.024**	**0.034**	**0.000**	**0.002**	**0.000**	**0.016**	0.657			
	*N*	**137**	**137**	**137**	**137**	**137**	**137**	**136**	137	137		
Depression	Coefficient	0.061	0.095	0.043	**0.279**	0.097	**0.370**	**0.332**	0.139	**0.567**	1	
	*p*-value	0.482	0.270	0.616	**0.001**	0.259	**0.000**	**0.000**	0.104	**0.000**		
	*N*	137	137	137	**137**	137	**137**	**136**	137	**137**	137	
HADS	Coefficient	0.137	**0.204**	0.128	**0.342**	**0.202**	**0.387**	**0.270**	0.056	**0.889**	**0.881**	1
	*p*-value	0.110	**0.017**	0.136	**0.000**	**0.018**	**0.000**	**0.001**	0.519	**0.000**	**0.000**	
	*N*	137	**137**	137	**137**	**137**	**137**	**136**	137	**137**	**137**	137
Chronotype	Coefficient	**0.201**	**−0.224**	−0.084	−0.056	**−0.239**	**−0.171**	−0.134	−0.086	0.086	0.077	0.092
	*p*-value	**0.019**	**0.008**	0.329	0.518	**0.005**	**0.046**	0.120	0.320	0.315	0.372	0.283
	*N*	**137**	**137**	137	137	**137**	**137**	136	137	137	137	137

CR, cognitive restraint; UE, uncontrolled eating; EE, emotional eating. Spearman and Pearson tests were used.

*Bold mean that the results are significant*.

**Table 7:** Correlations observed between the three aspects of eating habits and impulsivity, chronotype, anxiety, and depression among female participants.

		**CR**	**UE**	**EE**	**Negative urgency**	**Positive urgency**	**Lack of premeditation**	**Lack of perseverance**	**Seeking sensation**	**Anxiety**	**Depression**	**HADS**
Negative urgency	Coefficient	**−0.205**	**0.280**	**0.322**	1							
	*p*-value	**0.001**	**0.000**	**0.000**								
	N	**263**	**263**	**263**	263							
Positive urgency	Coefficient	**−0.260**	**0.176**	**0.246**	**0.608**	1						
	*p*-value	**0.000**	**0.004**	**0.000**	**0.000**							
	N	**263**	**263**	**263**	**263**	263						
Lack of premeditation	Coefficient	**−0.196**	**0.147**	**0.159**	**0.428**	**0.401**	1					
	*p*-value	**0.001**	**0.017**	**0.010**	**0.000**	**0.000**						
	N	**263**	**263**	**263**	**263**	**263**	263					
Lack of perseverance	Coefficient	**−0.162**	0.051	0.045	**0.215**	0.114	**0.520**	1				
	*p*-value	**0.008**	0.408	0.471	**0.000**	0.066	**0.000**					
	N	**263**	263	263	**263**	263	**263**	263				
Seeking sensations	Coefficient	0.048	0.097	**0.137**	**0.174**	**0.290**	0.064	**−0.163**	1			
	*p*-value	0.437	0.115	**0.026**	**0.005**	**0.000**	0.302	**0.008**				
	N	263	263	**263**	**263**	**263**	263	**263**	263			
Anxiety	Coefficient	−0.108	0.036	0.023	**0.274**	**0.238**	**0.163**	**0.149**	−0.032	1		
	*p*-value	0.080	0.560	0.706	**0.000**	**0.000**	**0.008**	**0.016**	0.610			
	N	263	263	263	**263**	**263**	**263**	**263**	263	263		
Depression	Coefficient	**−0.144**	0.095	0.071	**0.174**	0.119	**0.189**	**0.267**	**−0.124**	**0.545**	1	
	*p*-value	**0.019**	0.124	0.249	**0.005**	0.053	**0.002**	**0.000**	**0.044**	**0.000**		
	N	**263**	263	263	**263**	263	**263**	**263**	**263**	**263**	263	
HADS	Coefficient	**−0.141**	0.071	0.051	**0.260**	**0.210**	**0.199**	**0.208**	−0.079	**0.904**	**0.851**	1
	*p*-value	**0.022**	0.250	0.410	**0.000**	**0.001**	**0.001**	**0.001**	0.203	**0.000**	**0.000**	
	N	**263**	263	263	**263**	**263**	**263**	**263**	263	**263**	**263**	263
Chronotype	Coefficient	0.099	−0.061	**0.122**	0.008	0.038	−0.112	−0.012	−0.018	0.013	−0.041	−0.013
	*p*-value	0.111	0.327	**0.049**	0.903	0.544	0.071	0.844	0.775	0.835	0.504	0.833
	N	263	263	**263**	263	263	263	263	263	263	263	263

CR, cognitive restraint; UE, uncontrolled eating; EE, emotional eating. Spearman and Pearson tests were used.

*Bold mean that the results are significant*.

### Multivariate Analysis (Table 8)

Among females, increased BMI was associated to uncontrolled eating and emotional eating, while in males, BMI was associated to emotional eating only. In males, no associations of BMI with impulsivity were found while in females they were present.

**Table 8:** Multivariate analysis: factors significantly correlated to the three domains of the eating habits questionnaire.

	**Cognitive restraint**	**Non-standardized coefficients**		**Standardized coefficients**	***T***	***p*-value**	**Correlation**
		***B***	**Standard error**	**Beta**			
Females	BMI	0.145	0.078	0.114	1.873	0.062	0.117
	Negative urgency	−0.164	0.079	−0.138	−2.076	0.039	−0.129
	Lack of perseverance	−0.194	0.080	−0.151	−2.429	0.016	−0.150
	Depression	−0.144	0.071	−0.126	−2.036	0.043	−0.126
	Chronotype	0.032	0.023	0.085	1.396	0.164	0.087
	Depression^*^negative urgency	0.044	0.216	0.013	0.206	0.837	0.013
	Depression^*^lack of perseverance	0.126	0.223	0.035	0.566	0.572	0.035
Males	BMI	0.102	0.092	0.094	1.104	0.271	0.096
	Coffee	0.323	0.254	0.107	1.274	0.205	0.110
	Anxiety	0.117	0.072	0.138	1.619	0.108	0.140
	Chronotype	0.061	0.030	0.174	2.078	0.040	0.178
	**Uncontrolled eating**	**Non-standardized coefficients**		**Standardized coefficients**	***T***	***p*****-value**	**Correlation**
		***B***	**Standard error**	**Beta**			
Females	BMI	0.337	0.117	0.172	2.891	0.004	0.178
	Negative urgency	0.445	0.120	0.244	3.700	0.000	0.226
	Lack of premeditation	0.077	0.129	0.039	0.594	0.553	0.037
	Seeking sensations	0.099	0.106	0.056	0.936	0.350	0.059
	Depression	0.025	0.109	0.014	0.226	0.822	0.014
	Depression^*^negative urgency	0.329	0.368	0.063	0.893	0.373	0.056
	Depression^*^lack of premeditation	0.181	0.326	0.038	0.556	0.579	0.035
	Depression^*^seeking sensation	−0.016	0.332	−0.003	−0.047	0.962	−0.003
Males	Age	−0.650	0.253	−0.213	−2.570	0.011	−0.224
	BMI	0.259	0.148	0.145	1.757	0.081	0.155
	Alcohol	0.993	0.503	0.166	1.975	0.050	0.173
	Coffee	0.529	0.666	0.066	0.794	0.429	0.071
	Seeking sensations	0.174	0.152	0.093	1.142	0.256	0.102
	Anxiety	0.293	0.117	0.207	2.492	0.014	0.218
	Chronotype	−0.101	0.049	−0.172	−2.075	0.040	−0.182
	Anxiety^*^seeking sensations	−0.686	0.538	−0.123	−1.276	0.204	−0.110
	**Emotional eating**	**Non-standardized coefficients**		**Standardized coefficients**	***t***	***p***	**Correlation**
		***B***	**Standard error**	**Beta**			
Females	Age	0.109	0.081	0.078	1.341	0.181	0.084
	CI	0.516	0.370	0.081	1.395	0.164	0.087
	BMI	0.205	0.053	0.225	3.904	0.000	0.238
	Negative urgency	0.229	0.054	0.270	4.253	0.000	0.258
	Lack of premeditation	0.023	0.058	0.025	0.391	0.696	0.025
	Sensation seeking	0.083	0.047	0.101	1.749	0.082	0.109
	Chronotype	0.016	0.016	0.058	0.986	0.325	0.062
Males	BMI	0.134	0.066	0.171	2.032	0.044	0.173
	Negative urgency	0.103	0.069	0.131	1.484	0.140	0.128
	Anxiety	0.068	0.055	0.111	1.241	0.217	0.107
	Anxiety^*^ Negative urgency	0.084	0.134	0.037	0.628	0.530	0.026

* Means interaction (statistical interaction between both variables). *Gray mean that the results are significant*.

Chronotype scores were positively correlated to cognitive restraint and negatively to uncontrolled eating among males. No associations were found for females.

CR was lower among females with higher depression scores, while higher anxiety scores were associated to UE among males.

## Discussion

The results of this study showed a gender difference in the emotional eating (EE) component (higher in females). This finding confirms previously published results showing that females turn out to be more emotional eaters than males (Hantsoo and Epperson, 2017). Males resort less to emotional eating to overcome their negative feelings, they rather resort to other ways of coping such as gambling, alcohol drinking or internet addiction (Asarian and Geary, 2013).

Significant associations between two dimensions of eating habits (uncontrolled eating UE and EE) and BMI were observed. Thus, an increased BMI was associated to emotional eating in both genders. This result was further supported by the finding that emotional eating scores increased steadily from underweight to obese categories (in both genders).

For UE, it was associated to increased BMI only in females and when BMI was analyzed as a continuous variable, not as categorized. The findings show that UE and EE may be correlated to an increase in BMI was already reported in previous studies. What was interesting in this study were the gender specificities observed highlighting EE as principal component related to BMI in both genders while UE association to BMI was only significant in females. CR was not significantly associated to BMI in our study (analyzed as continuous variable). The only significance observed was among females after categorizing the BMI: normal weight females scored the highest on cognitive restraint component. Cognitive restraint (CR) is the intention to control food intake in order to maintain or lose weight (Hofmann et al., 2014; Julien Sweerts et al., 2019). The impact of CR on weight is very controversial; Many studies have shown a correlation between weight or body mass index (BMI) and CR, either negative (de Lauzon-Guillain et al., 2017; Singh et al., 2017) or positive (Banna et al., 2018).

UE was inversely associated to age and positively to alcohol intake among males, highlighting the fact that when age increases, uncontrolled eating decreases and that participants with larger alcohol intake had also higher UE behavior.

Interestingly, impulsivity was intimately associated to eating habits in females as well as depression to lower cognitive restraint while chronotype and anxiety were two factors associated to eating habits domains in males. Higher negative urgency and lack of perseverance were associated to lower CR among females while UE and EE were both associated to higher negative urgency. High impulsivity was already reported to be associated with personality disorders in bulimia nervosa and the inverse was observed in anorexia nervosa (Cassin and von Ranson, 2005). In addition, previous studies have pointed to similarities between addictive behaviors and eating disorders and both individuals with addiction and restrained eating behavior presented higher impulsivity (Jansen et al., 1989). Negative urgency is defined as the propensity to act out when experiencing negative emotions and it was already linked to substance use disorders and eating disorders (Owens et al., 2018). It could be a positive moderator of reactivity to stressful situations (Owens et al., 2018). Negative urgency was associated with eating concern and frequency of loss of control over eating (Lavender et al., 2017) and predicted binge eating and weight and shape concerns (Stojek et al., 2014). This is the first study showing that eating behavior in females, impulsivity in general and negative urgency in particular are significantly associated. These associations were not significant among males.

Another novel finding in this study was the association between eating habits and the chronotype among males: morning type individuals had higher CR and lower UE. A single precedent study found a positive association between morningness and dietary restraint (Schubert and Randler, 2008). More is known about links between food addiction and evening type individuals who seem more likely to exhibit food addiction than the morning types (Kandeger et al., 2018).

Finally, correlations between eating habits and anxiety or depression show gender differences. While depression scores were inversely associated to CR among females (an increase in depression score was associated to lower CR), anxiety increases in parallel to UE among males.

Depression was previously reported to be a major consequence of being overweight (Barnes et al., 2015). In addition, anxiety and depression can lead to an overconsumption of food as means to cope, leading to food consumption (Yau and Potenza, 2013), mostly comfort food (Andersen et al., 2010; Boutelle et al., 2010). Our study cannot establish a causal relationship between depression or anxiety and eating behavior because of the cross-sectional design.

Our finding that anxiety is associated to UE among males confirms previous results that uncontrolled eating (but not emotional eating or cognitive restraint) significantly mediated a relationship between certain type of anxiety and BMI (Wilkinson et al., 2019). Previous studies revealed that depression history and severity were associated with less cognitive restrained eating (Paans et al., 2018), which is similar to our results. However, gender differences were not reported before.

Our findings should take into account the study's design and limitations. The results were obtained through self-reported questionnaires. Even though self-reporting questionnaires are widely used in community surveys (NIMH; Ciarma and Mathew, 2017), the self-report methods reflect the participant's own perspective, and could lead to bias due to forgetting or not willing to disclose information sometimes. The questionnaires were formulated as scales or multiple-choice to make it easier to respond and to shorten the interview duration as much as possible, thus avoiding to disturb the students. The simplicity of the questionnaire makes it somewhat easy for the participants to give accurate information. Another limitation is that neither insomnia nor sleep quality have been examined and thus need to be explored in future studies since they are important determinant of eating habits. Chronic disease and chronic medications were among the exclusion criteria, therefore, the impact of comorbidities or the use of drugs were not examined. Another limitation is that the sample is imbalanced for male and female ratio; even though the ratio was balanced during the random selection (56% females vs. 44% males, similar to the gender distribution of the university students), 81% of the females approached agreed to participate vs. only 53.7% of the males that we approached. This limitation was taken into account in part by separating females' data from males.

Finally, our study cannot establish a causal relationship between impulsivity, depression or anxiety and eating behavior because of the cross-sectional design. Other, prospective longitudinal studies are needed to establish causality with eating behavior.

Despite the limitations, several findings observed in this study are of major importance in explaining eating habits and their relation to BMI and warrant further future investigations.

To the best of our knowledge, this study was the first to assess the relationships between four different neuropsychiatric aspects, i.e., impulsivity, chronotype, anxiety, and depression, and eating habits among young adults. Even though some findings were already reported in other countries, our results are very informative for the Lebanese population.

In conclusion, this was a cross-sectional study of three cognitive and emotional domains related to eating habits among university students (young adults). We surveyed chronotype, impulsivity and affective scores (depression/anxiety). Results showed significant correlations between BMI, TFEQ-R18 scores, impulsivity and anxiety or depression. There was a significant association between two dimensions of eating habits (UE and EE) and BMI. Significant correlations between eating habits and impulsivity domains or depression were observed among females. Among males, chronotype and anxiety seem to play a key role. Future studies should replicate findings in samples of individuals with different aspects of eating disorders such as binge eating disorder, food addiction or bulimia nervosa.

## Data Availability

The raw data supporting the conclusions of this manuscript will be made available by the authors, without undue reservation, to any qualified researcher.

## Ethics Statement

The protocol of the study was approved by the ethics committee of Saint-Joseph University (Ref USJ-2017-81). Informed written consent was obtained from all individuals participating in the study.

## Author Contributions

CA, LN, and SS: field work, entering results, double checking data. NE: statistical analysis. TP: writing the manuscript draft. LR: supervising the study and writing the final version of the manuscript.

### Conflict of Interest Statement

The authors declare that the research was conducted in the absence of any commercial or financial relationships that could be construed as a potential conflict of interest.
